# Relationship between creatinine to body weight ratios and diabetes mellitus: A Chinese cohort study

**DOI:** 10.1111/1753-0407.13248

**Published:** 2022-01-10

**Authors:** Zhuangsen Chen, Yang Zou, Fan Yang, Xiao han Ding, Changchun Cao, Haofei Hu, Xinyu Wang

**Affiliations:** ^1^ Department of Endocrinology Pingshan District People's Hospital of Shenzhen Shenzhen China; ^2^ Pingshan General Hospital of Southern Medical University Shenzhen China; ^3^ Department of Cardiology Jiangxi Provincial People's Hospital Affiliated to Nanchang University Nanchang China; ^4^ Jiangxi Cardiovascular Research Institute Nanchang China; ^5^ Department of Endocrinology Shenzhen Second People's Hospital Shenzhen China; ^6^ Shenzhen University Health Science Center Shenzhen China; ^7^ Department of Rehabilitation Shenzhen Dapeng New District Nan'ao People's Hospital Shenzhen China; ^8^ Department of Nephrology Shenzhen Second People's Hospital Shenzhen China

**Keywords:** body weight, creatinine, diabetes incident, dummy variables, multiple imputations, nonlinearity, 肌酐, 体重, 糖尿病发病率, 多重插补, 哑变量, 非线性。

## Abstract

**Background:**

Research on the relationship between creatinine to body weight ratios (Cre/BW ratios) and the prevalence of diabetes is still lacking. The intention of this research was to explore the potential relationship between Cre/BW ratio and diabetes prevalence in Chinese adults.

**Methods:**

This retrospective study was conducted on 199 526 patients in the Chinese Rich Healthcare Group from 2010 to 2016. The participants were divided into four groups on the basis of the quartiles of the Cre/BW ratios. Multivariate multiple imputation and dummy variables were used to handle missing values. Multivariate regression analysis was applied to detect the relationship between Cre/BW and diabetes. A smoothing plot was also used to identify whether there were nonlinear relationships.

**Results:**

After handling missing values and adjusting for potential confounders, the multivariate Cox regression analysis results showed that Cre/BW was inversely correlated with diabetes risk (hazard ratio [HR]: 0.268; 95% confidence interval [CI]: 0.229–0.314, *p* < 0.00001). For men, the HR of incident diabetes was 0.255 (95% CI: 0.212–0.307) and for women it was 0.297 (95% CI: 0.218–0.406). Moreover, sensitivity analysis confirmed the stability of the results. Furthermore, the smoothing plot revealed that there was a saturation effect between Cre/BW and the incidence of diabetes.

**Conclusions:**

This study demonstrated that increased Cre/BW is negatively correlated with diabetes in Chinese adults. It also found that Cre/BW has a nonlinear relationship with the incidence of diabetes.

## Graphical abstract

**Highlights** In Chinese adults, creatinine to body weight ratio is inversely associated with diabetes risk, and the relationship is nonlinear. Those who have diabetes risk factors and have low creatinine to body weight ratio should be vigilant against the occurrence of diabetes.

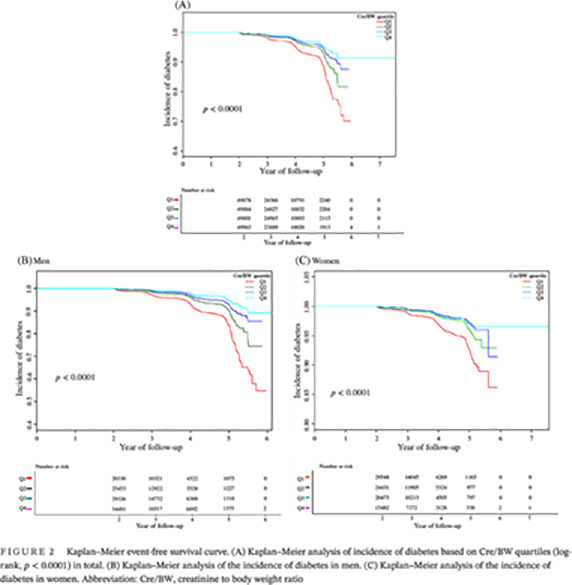

## 摘要

**背景:**

肌酐/体重比值(Cre/BW)与糖尿病患病率的关系尚缺乏研究。本研究旨在探讨CRE/BW比值与中国成年人糖尿病患病率之间的潜在关系。

**方法:**

本回顾性队列研究纳入中国华富医疗项目(Chinese Rich Healthcare Group)从2010 ‐2016年的199,526例患者。根据Cre/BW比率的四分位数，参与者被分成四组。应用多重插补及哑变量处理缺失值。采用多元回归分析探讨Cre/BW与糖尿病发病风险之间的关系，并运用平滑拟合曲线分析两者是否存在非线性关系。

**结果:**

在所有受试者中，在处理缺失值和校正潜在混杂因素后，多元回归分析结果显示，Cre/BW与糖尿病风险呈负相关(HR:0.268；95%CI:0.229~0.314，P<0.00001)；男性发生糖尿病的危险比(HR)为0.255(95%CI:0.212~0.307)；女性为0.297(95%CI:0.218~0.406)，敏感性分析证实了结果的稳定性。此外，平滑拟合曲线分析显示CRE/BW与糖尿病发病率之间存在饱和效应。

**结论:**

本研究表明中国人Cre/BW增加与糖尿病呈负相关。研究还发现，Cre/BW与糖尿病发病率之间存在非线性关系。

## 1 INTRODUCTION

Diabetes mellitus (DM) is an important issue concerning the health of the whole population, which contributes to the global health burden. In 2019, there were 351.7 million people (ages 20–64) with diagnosed or undiagnosed diabetes. It is estimated that by 2045, this number will increase to 486.1 million.^1^ In the future, the aging of the population will further increase the number of people with diabetes and bring greater challenges to public health and the economy.^2^, ^3^ Besides being reported in adults, type 2 diabetes (T2DM) is also increasing in children and adolescents, leading to early complications and serious adverse health consequences.^4^ Therefore, it is essential to identify the occurrence of diabetes early in order to establish preventive strategies for this disease.

Skeletal muscle is one of the main target organs of insulin, which plays a significant role in maintaining glucose homeostasis.^5^, ^6^ Decrease in the skeletal muscle mass leads to a reduction in systemic glucose uptake.^7^ It contributes to insulin resistance both in nonobese and obese individuals.^8^, ^9^ Skeletal muscle mass is related to many diseases, such as insulin resistance, DM, metabolic syndrome (MS), nonalcoholic fatty liver (NAFLD), and cardiovascular disease.^10^, ^11^, ^12^ Insulin can enhance the synthesis of muscle protein and inhibit the breakdown of muscle protein. Both insulin resistance and insulin deficiency can cause a decrease in insulin signals in the skeletal muscle, affect the regulation of skeletal muscle protein balance, and may cause a decline in skeletal muscle quality.^11^ Abnormal muscle protein metabolisms and skeletal muscle atrophy have been observed in patients with T2DM.^7^, ^13^ Therefore, the decrease of skeletal muscle mass is related to the occurrence of insulin resistance and diabetes.

Serum creatinine (Cre) is considered to be an inexpensive and easy‐to‐measure index instead of evaluating skeletal muscle quality.^14^ Recently, studies have shown that Cre to body weight ratios (Cre/BW), an interesting new indicator, is closely related to T2DM^15^ and NAFLD.^16^, ^17^ Hashimoto et al. proved Cre/BW ratios may predict the risk of diabetes and are negatively interrelated with the incidence of diabetes in the Japanese population who underwent a health checkup.^15^ However, there is no report about Cre/BW ratios with diabetes to date in Chinese people. To address these issues, the motivation of this study was to detect the relationship between Cre/BW and diabetes in Chinese people.

## 2 METHODS

### 2.1 Study population and design

The present data were acquired in the public database DATADRYAD (www.Datadryad.org), published by Chen et al.^18^ Allowing a no charge download, all the copyright and the ownership of the data in the website have been given up by the providers. Previous studies have been approved by Rich Healthcare Group Review Committees and conducted a retrospective analysis of the information based on the Helsinki Declaration.^18^ This study is also a retrospective observational study. The original research did not provide informed consent. The research recruited 685 277 participants over 20 years old who had accepted at least two health checkups in China from 2010 to 2016. The data used in this study have been initially screened, and 211 833 participants were included in the analysis. The research design and selection criteria have been described in detail in a previous article.^18^ For our further research, Cre/BW ratios was calculated as Cre divided by body weight, in which missing values (n = 11 175) and outliers of Cre/BW ratios (<means minus 3 SD [SD] or > means plus 3 SD) (n = 1132) were excluded.^19^ Ultimately, this retrospective study was conducted in 199 526 participants (109 590 male and 89 936 female).

### 2.2 Data Collection and Measurements

The researchers collected and measured the study cohort's information and described it in detail previously.^18^, ^20^ Briefly, questionnaires were administered to collect information on demographics (age, sex), lifestyle (smoking, alcohol consumption), family history of disease, and personal medical history in each visit. BW and height of all participants were measured in light clothing without shoes on. Body mass index (BMI) was obtained from the formula weight (kg) divided by the square of height (meters). Parameters of blood pressure (BP) were measured with a mercury manometer. Smoking status was classified as quitters, current smoker, and never smoker. Drinking status was classified as former drinker, current drinker, and never drinker. Venous blood was drawn after fasting for at least 10 h at each visit and measured on autoanalyzers for fasting plasma glucose (FPG), triglyceride (TG), total cholesterol (TC), low‐density lipoprotein cholesterol (LDL‐C), high‐density lipoprotein cholesterol (HDL‐C), and Cre. FPG of ≥7.00 mmol/L and/or self‐reported diabetes during the follow‐up was defined as the incidence of diabetes.

### 2.3 Statistical analysis

Statistical analyses with the outcomes were run in the statistical software package R (http://www.R-project.org, The R Foundation) and Empower‐Stats (http://www.empowerstats.com, X&Y Solutions, Inc., Boston, MA). First, the patients were divided based on the quartiles of baseline Cre/BW ratios. Continuous variables were reported by mean ± SD and the Student *t*‐test was used for comparison, the categorical variables were reported by frequency (percentage), and comparison was performed by chi‐square test. Subsequently, multiple linear regression analysis was used to calculate the variance inflation factor to check the collinearity of variables.^21^ The risk of Cre/BW ratios on diabetes was assessed by calculating the hazard ratio (HR) and 95% confidence interval (CI) using Cox proportional‐hazards model and adjusted for age, gender, systolic BP (SBP), diastolic BP (DBP), FPG, TG, HDL‐C, LDL‐C, smoking consumption, drinking consumption, and family history of diabetes. Unadjusted and adjusted HRs and 95% CIs were used to quantify and assess the strength of the association. When added to the model, variables with a matching risk rate change of more than 10% were adjusted..^22^ Based on the proposal of the Strengthening the Reporting of Observational Studies in Epidemiology (STROBE statement), all of the models would be adjusted for none (model I), age, gender, SBP, DBP, smoking consumption, alcohol consumption, and family history of diabetes (model II), model2+ FPG, TG, HDL‐C, and LDL‐C (model III).^22^ The Cre/BW was transformed into a categorical variable to calculate the *P* value of trend, used to verify the result of Cre/BW as a continuous variable, and the possibility of nonlinearity was observed. Besides, considering the limitations of the generalized linear model when handing the nonlinearity, the weighted generalized additive model (GAM)^23^ was used in adjustment of the covariates in the GAM model.

In addition, in order to maximize statistical power and reduce bias, this study used the following analysis to deal with missing values of covariates. When the missing data were <20%, the multiple imputations of the chain equation^24^, ^25^, ^26^ were used to estimate the missing value; otherwise, dummy variables^27^ were used to indicate missing continuous variables. The missing values of the categorical variables^27^, ^28^ were treated as a new categorical variable group. The original cohort data (included only patients with no missing value in all variables) were repeated baseline and Cox proportional hazards regression analysis as a sensitivity analysis.

Next, a smoothing plot was also used to observe the relationship between the Cre/BW and diabetes risk.^29^ If there was a nonlinear correlation in the smoothing plot, the threshold effect was investigated by using two‐piecewise linear regression model. The inflection point was automatically calculated by recursive method and then applied with maximum likelihood model when the ratio of Cre/BW to diabetes risk was evident in the smooth curve. Additionally, Kaplan‐Meier analysis and logarithmic rank test were also carried out to compare the discrepancy between Cre/BW quartiles. Furthermore, considering the potential effects of sex in the Cre/BW ratios, the study separately analyzed men and women. To investigate the robustness of the results of different groups of age, data were analyzed using Cox proportional risk model. The model was adjusted for gender, SBP, DBP, FPG, TG, HDL‐C, LDL‐C, smoking and drinking status, and family history of diabetes. The likelihood ratio was used to test the modification and interaction of subgroups.

On the other hand, in order to further evaluate the predictive performance of Cre/BW, BMI, weight, TG, HDL‐C, and Cre for diabetes, we also plotted the receiver operating characteristic (ROC) curve and calculated the area under the curve (AUC) of each parameter for predicting diabetes.

*p* < 0.05 on both sides was statistically significant.

## 3 RESULTS

Ultimately, a total of 199 526 participants (109 590 male and 89 936 female) were included in our analysis. The average follow‐up was 3.13 ± 0.94 years, and the mean Cre/BW was 1.09 ± 0.22. The mean age of males was 42.34 ± 12.87 years old, and the average age of females was 41.99 ± 12.38 years old. The mean Cre levels in men and women were 79.67 ± 11.30 and 57.84 ± 8.98 umol/L, and the mean BMI numbers were 24.22 ± 3.23 and 22.09 ± 3.08 kg/m^2^, respectively. At the end of the study, 2872 men and 1103 women were newly diagnosed with diabetes.

### 3.1 Baseline characteristics of the study participants

Table 1 summarized baseline characteristics of original data. According to Cre/BW quartiles, participants were divided into four subgroups: quartile 1 (Q1), Cre/BW < 0.94; quartile 2 (Q2), 0.94 ≤ Cre/BW <1.08; quartile 3 (Q3), 1.08 ≤ Cre/BW <1.23, and quartile 4 (Q4) Cre/BW >1.23. The incidence of diabetes in each quartile was 3.230%, 1.995%, 1.542%, and 1.203%, respectively. In the lowest Cre/BW group, the study found that participants had higher BMI, SBP, DBP, FPG, TC, and LDL‐C and lower Cre. Moreover, (Table S1) listed the baseline characteristics of males and females, respectively. There are significant differences in the smoking consumption and alcohol consumption of different groups of men; however, no significant differences were found in women.

**TABLE 1:** Baseline characteristics of participants according to the quartiles of Cre/BW ratios

Cre/BW	Q1 (≤0.94)	Q2 (0.94 to ≤1.08)	Q3 (1.08 to ≤1.23)	Q4 (>1.23)	*p* value
Age (years)	42.37 ± 11.69	42.12 ± 12.04	41.97 ± 12.65	42.27 ± 14.09	<0.001
Gender					<0.001
Male	20 330 (40.76%)	25 453 (51.02%)	29 326 (58.89%)	34 481 (69.01%)	
Female	29 548 (59.24%)	24 431 (48.98%)	20 475 (41.11%)	15 482 (30.99%)	
Height (cm)	166.61 ± 8.68	166.63 ± 8.46	166.62 ± 8.23	166.21 ± 7.90	<0.001
Weight (kg)	71.00 ± 13.54	65.78 ± 11.74	63.11 ± 10.73	59.34 ± 9.40	<0.001
BMI (kg/m^2^)	25.43 ± 3.50	23.56 ± 2.95	22.63 ± 2.80	21.42 ± 2.66	<0.001
SBP (mm Hg)	121.38 ± 16.81	118.80 ± 16.26	118.02 ± 15.89	117.84 ± 16.27	<0.001
DBP (mm Hg)	75.65 ± 11.32	74.20 ± 10.83	73.70 ± 10.55	73.08 ± 10.31	<0.001
FPG (mmol/L)	4.79 ± 0.92	4.73 ± 0.90	4.70 ± 0.89	4.64 ± 0.88	<0.001
TC (mmol/L)	4.99 ± 0.62	4.92 ± 0.61	4.88 ± 0.61	4.87 ± 0.61	<0.001
TG (mmol/L)	1.51 ± 1.23	1.37 ± 1.04	1.30 ± 0.96	1.19 ± 0.84	<0.001
HDL‐C (mmol/L)	1.34 ± 0.31	1.37 ± 0.31	1.38 ± 0.31	1.39 ± 0.30	<0.001
LDL‐C (mmol/L)	2.81 ± 0.70	2.77 ± 0.68	2.76 ± 0.68	2.73 ± 0.66	<0.001
Cre (umol/L)	58.59 ± 11.38	66.34 ± 11.94	72.49 ± 12.41	81.90 ± 13.44	<0.001
Smoking status					<0.001
Never smoker	10 453 (78.22%)	10 863 (76.18%)	10 942 (75.16%)	11 284 (74.59%)	
Ever smoker	505 (3.78%)	590 (4.14%)	673 (4.62%)	691 (4.57%)	
Current smoker	2405 (18.00%)	2807 (19.68%)	2943 (20.22%)	3154 (20.85%)	
Drinking status					<0.001
Never drinker	11 313 (84.66%)	11 826 (82.93%)	11 865 (81.50%)	12 412 (82.04%)	
Ever drinker	1758 (13.16%)	2094 (14.68%)	2341 (16.08%)	2420 (16.00%)	
Current drinker	292 (2.19%)	340 (2.38%)	352 (2.42%)	297 (1.96%)	
Family history of diabetes					<0.001
No	48 504 (97.25%)	48 783 (97.79%)	48 842 (98.07%)	49 205 (98.48%)	
Yes	1374 (2.75%)	1101 (2.21%)	959 (1.93%)	758 (1.52%)	
incidence of diabetes	1611 (3.230%)	995 (1.995%)	768 (1.542%)	601 (1.203%)	<0.001

*Note*: Values are n(%) or mean ± SD.

Abbreviations: BMI, body mass index; Cre, serum creatinine; Cre/BW, creatinine to body weight ratio; DBP, diastolic blood pressure; FPG, fasting plasma glucose; HDL‐C, high‐density lipoprotein cholesterol; LDL‐C, low‐density lipid cholesterol; sBP, Systolic blood pressure; TC, total cholesterol; TG, triglyceride.

### 3.2 The processing of missing values

The data set contained 20 missing values of SBP, 21 of DBP, 3102 of TG (accounting for 3.93% of the total data), 84 309 of HDL‐C and LDL‐C (43.3%, respectively), 142 216 of smoking and alcohol consumption (71.3%, respectively). (listed in Table S2). Sensitivity analysis on the original and complete‐case data (Table S3) found that there were statistical differences between the original data and the complete‐case data (*p* < 0.05). In order to assess the impact of the bias caused by the missing data, the following analysis was performed in the study. Researchers respectively treated missing data of smoking and drinking status as a categorical variable and used multiple imputations for missing data of SBP, DBP, TG, HDL‐C, and LDL‐C, based on five replications and a chained equation approach method (Table S4). Interestingly, after repeated sensitivity analysis, there was still a distinct difference in HDL‐C and LDL‐C between preimputation and postimputation (*p* < 0.001). Thus, after multiple imputations at SBP, DBP, and TG, the missing values for HDL‐C and LDL‐C were estimated by dummy variables. Moreover, the missing values of smoking and drinking status were respectively regarded as a new classified variable group.

### 3.3 The multivariate analysis of Cre/BW with DM risk

First, researchers conducted screening variables collinearity diagnostics, and the results and details are presented in (Table S5). Second, the results of multivariate regression analysis after missing value processing are shown in Table 2 (Queue I). In the crude model, Cre/BW was negatively associated with the incidence of diabetes (HR: 0.095, 95% CI: 0.081–0.111, *p* < 0.00001). In Model I (adjusted age, gender, family history of diabetes, smoking and drinking consumption), the conclusion was the same as the crude model (HR: 0.092, 95% CI: 0.078–0.108). After adjusted age, gender, SBP, DBP, FPG, TG, HDL‐C, LDL‐C, smoking and drinking consumption, and family history of diabetes (Model II), the results remained negative (HR: 0.268, 95% CI: 0.229–0.314). A significant relationship between the Cre/BW and the risk of incident diabetes was found in both basic and fully adjusted models, with a HR of 0.790 (95% CI: 0.777–0.803) and 0.877 (95% CI: 0.863–0.891) reported for per–0.10 Cre/BW increase. Sensitivity analysis using Cre/BW as a categorical variable (quartile) found that in Model II, the risk of diabetes was 53.9% lower in the top quartile than in the bottom quartile, and significant trends were found in the quartile (*P* for trend<0.00001). Then the continuous covariates were inserted into the equation in the form of curves by GAM. In GAM model, the results are roughly the same as before (HR: 0.314; 95% CI: 0.226–0.369, *p* < 0.00001), showing the robustness of the results. Third, multiple regression analysis was used to compare the relationship between Cre/BW and diabetes risk under different missing processing modes (Queue II and Queue III in Table 2). The result retained a negative correlation between Cre/BW and the incidence of diabetes under different modes. In addition, when the covariates are not adjusted (crude model), the HRs of incident diabetes in the mode where multiple and dummy variables were used to estimate the missing values of the continuous variable (Queue I) was the same as the HRs for complete‐case data (Queue II). It seems that the result is more reliable.

**TABLE 2:** Relationship between Cre/BW and the incidence of diabetes in different models

Queue		Crude model (HR, 5% CI, *p*)	Model I (HR, 95% CI, *p*)	Model II (HR, 95% CI, *p*)	GAM (HR, 95% CI, *p*)
Queue I	Cre/BW	0.095 (0.081, 0.111) <0.00001	0.092 (0.078, 0.108) <0.00001	0.268 (0.229, 0.314) <0.00001	0.314 (0.266, 0.369) <0.00001
	Per–0.10 increase	0.790 (0.777, 0.803) <0.00001	0.788 (0.775, 0.801) <0.00001	0.877 (0.863, 0.891) <0.00001	0.891 (0.876, 0.905) <0.00001
	Cre/BW (quartile)				
	Q1	Ref	Ref	Ref	Ref
	Q2	0.565 (0.522, 0.612) <0.00001	0.585 (0.540, 0.634) <0.00001	0.711 (0.656, 0.770) <0.00001	0.724 (0.668, 0.784) <0.00001
	Q3	0.413 (0.379, 0.451) <0.00001	0.431 (0.395, 0.471) <0.00001	0.622 (0.570, 0.680) <0.00001	0.654 (0.598, 0.714) <0.00001
	Q4	0.317 (0.288, 0.348) <0.00001	0.303 (0.275, 0.334) <0.00001	0.506 (0.459, 0.558) <0.00001	0.554 (0.502, 0.612) <0.00001
	*p* for trend	<0.00001	<0.00001	<0.00001	<0.00001
Queue II	Cre/BW	0.095 (0.081, 0.111) <0.00001	0.066 (0.049, 0.090) <0.00001	0.182 (0.121, 0.273) <0.00001	0.201 (0.134, 0.302) <0.00001
	Per–0.10 increase	0.790 (0.777, 0.803) <0.00001	0.762 (0.739, 0.786) <0.00001	0.843 (0.810, 0.878) <0.00001	0.852 (0.818, 0.887) <0.00001
	Cre/BW (quartile)				
	Q1	Ref	Ref	Ref	Ref
	Q2	0.565 (0.522, 0.612) <0.00001	0.589 (0.508, 0.682) <0.00001	0.690 (0.571, 0.833) 0.00012	0.714 (0.590, 0.865) 0.00056
	Q3	0.413 (0.379, 0.451) <0.00001	0.376 (0.318, 0.444) <0.00001	0.529 (0.428, 0.655) <0.00001	0.552 (0.446, 0.685) <0.00001
	Q4	0.317 (0.288, 0.348) <0.00001	0.249 (0.206, 0.300) <0.00001	0.436 (0.340, 0.557) <0.00001	0.461 (0.359, 0.592) <0.00001
	*p* for trend	<0.00001	<0.00001	<0.00001	<0.00001
Queue III	Cre/BW	0.146 (0.124, 0.170) <0.00001	0.092 (0.078, 0.109) <0.00001	0.110 (0.094, 0.130) <0.00001	0.143 (0.121, 0.169) <0.00001
	Per–0.10 increase	0.825 (0.812, 0.838) <0.00001	0.788 (0.775, 0.801) <0.00001	0.802 (0.789, 0.815) <0.00001	0.824 (0.810, 0.837) <0.00001
	Cre/BW (quartile)				
	Q1	Ref	Ref	Ref	Ref
	Q2	0.613 (0.567,0.664) < 0.00001	0.585 (0.540,0.634) <0.00001	0.611 (0.564, 0.662) < 0.00001	0.631 (0.582, 0.683) < 0.00001
	Q3	0.476 (0.437, 0.518) < 0.00001	0.431 (0.395,0.471) <0.00001	0.461 (0.422,0.503) < 0.00001	0.494 (0.452,0.539) < 0.00001
	Q4	0.392 (0.357,0.431) < 0.00001	0.303 (0.275,0.334) <0.00001	0.335 (0.303,0.369) < 0.00001	0.394 (0.357, 0.435) < 0.00001
	*p* for trend	<0.00001	<0.00001	<0.00001	<0.00001

*Note*: Queue I: we handled missing data of smoking and drinking status as a categorical variable; used multiple imputations at SBP, DBP, and TG; and estimated HDL‐C and LDL‐C by dummy variables. Queue II: No missing value processing (a complete‐case analysis). Queue III: we handled missing data of smoking and drinking status as a categorical variable and used multiple imputations at SBP, DBP, TG, HDL‐C, and LDL‐C. Crude model: we did not adjust other covariants. Model I: we adjusted for age, gender, SBP, DBP, smoking status, drinking status, and family history of diabetes. Model II: we adjusted for age, gender, SBP, DBP, FPG, TG, HDL‐C, LDL‐C, smoking and drinking status, and family history of diabetes. GAM: All covariates listed in model II were adjusted. However, continuous covariates were adjusted as nonlinearity.

Abbreviations: CI, confidence interval; Cre/BW, creatinine to body weight ratio; DBP, diastolic blood pressure; FPG, fasting plasma glucose; GAM, generalized additive model; HDL‐C, high‐density lipoprotein cholesterol; HR, hazard ratio; LDL‐C, low‐density lipid cholesterol; Ref: reference; SBP, systolic blood pressure; TG, triglyceride.

### 3.4 Correlation between Cre/BW and the incidence of diabetes in men and women after missing value processing

Table 3 presented the results of Cox proportional‐hazards regression analyses between Cre/BW and the incidence of diabetes in men and women. After adjusting for age, gender, SBP, DBP, FPG, TG, HDL‐C, LDL‐C, smoking and drinking consumption, and family history of diabetes, Cre/BW was negatively correlated with diabetes risk for men (0.255; 95% CI: 0.212–0.307) and women (0.297; 95% CI: 0.218–0.406). Furthermore, it was also consistent with the GAM (HR: 0.295; 95% CI: 0.245 to 0.356 for men and HR: 0.340; 95% CI: 0.248 to 0.466 for women). In addition, when the quartile of Cre/BW was used as a categorical variable, the trend between quartiles was also statistically significant (*P* for trend <0.00001).

**TABLE 3:** Relationship between Cre/BW and the incidence of diabetes in men and women

Men	Crude model (HR, 95% CI, *p*)	Model I (HR, 95% CI, *p*)	Model II (HR, 95% CI, *p*)	GAM (HR, 95% CI, *p*)
Cre/BW	0.097 (0.081, 0.117) <0.00001	0.089 (0.074, 0.108) <0.00001	0.255 (0.212, 0.307) <0.00001	0.295 (0.245, 0.356) <0.00001
Cre/BW(quartile)				
Q1	Ref	Ref	Ref	Ref
Q2	0.572 (0.521, 0.629) <0.00001	0.570 (0.519, 0.627) <0.00001	0.698 (0.635, 0.768) <0.00001	0.710 (0.645, 0.780) <0.00001
Q3	0.417 (0.377, 0.461) <0.00001	0.420 (0.380, 0.465) <0.00001	0.610 (0.550, 0.675) <0.00001	0.639 (0.576, 0.708) <0.00001
Q4	0.295 (0.265, 0.329) <0.00001	0.283 (0.254, 0.316) <0.00001	0.472 (0.422, 0.528) <0.00001	0.514 (0.459, 0.575) <0.00001
*p* for trend	<0.00001	<0.00001	<0.00001	<0.00001

Women	Crude model (HR, 95% CI, *p*)	Model I (HR, 95% CI, *p*)	Model II (HR, 95% CI, *p*)	GAM (HR, 95% CI, *p*)
Cre/BW	0.088 (0.064, 0.121) <0.00001	0.103 (0.075, 0.140) <0.00001	0.297 (0.218, 0.406) <0.00001	0.340 (0.248, 0.466) <0.00001
Cre/BW(quartile)				
Q1	Ref	Ref	Ref	Ref
Q2	0.541 (0.467, 0.627) <0.00001	0.616 (0.531, 0.715) <0.00001	0.724 (0.623, 0.841) 0.00003	0.752 (0.647, 0.875) 0.00023
Q3	0.394 (0.330, 0.470) <0.00001	0.451 (0.378, 0.539) <0.00001	0.650 (0.542, 0.779) <0.00001	0.685 (0.570, 0.822) 0.00005
Q4	0.431 (0.355, 0.523) <0.00001	0.393 (0.322, 0.479) <0.00001	0.616 (0.503, 0.755) <0.00001	0.652 (0.529, 0.804) 0.00006
*p* for trend	<0.00001	<0.00001	<0.00001	<0.00001

*Note*: Crude model: we did not adjust other covariants. Model I: we adjusted for age, gender, SBP, DBP, smoking status, drinking status, and family history of diabetes. Model II: we adjusted for age, gender, SBP, DBP, FPG, TG, HDL‐C, LDL‐C, smoking and drinking status, and family history of diabetes. GAM: All covariates listed in model II were adjusted. However, continuous covariates were adjusted as nonlinearity.

Abbreviations: Cre/BW, creatinine to body weight ratio; CI, confidence interval; DBP, diastolic blood pressure; FPG, fasting plasma glucose; GAM, generalized additive model; HDL‐C, high‐density lipoprotein cholesterol; HR, hazard ratio; LDL‐C, low‐density lipid cholesterol; Ref, reference; SBP, systolic blood pressure; TG, triglyceride.

### 3.5 The result of the smoothing plot and threshold effect

**FIGURE 1:**
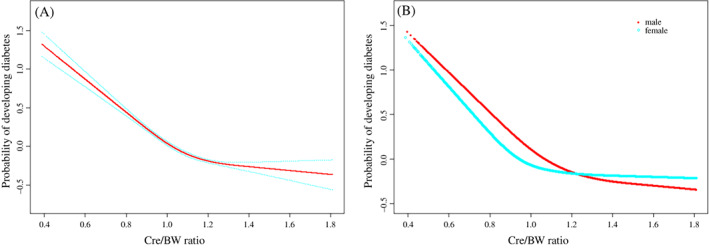
The nonlinear relationship between Cre/BW and incidence of diabetes (A), and a nonlinear relationship between them in different sexes (B). Abbreviation: Cre/BW, creatinine to body weight ratio

The smoothing plot was performed to explore the nonlinear relationship between Cre/BW and the incidence of diabetes (Figure 1), based on one replication of multiple imputation data. The study found a nonlinear relationship (after adjusting for age, gender, SBP, DBP, FPG, TG, HDL‐C, LDL‐C, smoking and drinking consumption, and family history of diabetes) in total (HR: 0.268; 95% CI: 0.229–0.314) and different sex group (HR: 0.255; 95% CI: 0.212–0.307 for men and HR: 0.297; 95% CI: 0.218–0.406 for women). The inflection point of Cre/BW calculated by the two‐piecewise linear regression model was 1.06 (log‐likelihood ratio test *p* < 0.001). On the left side of the inflection point, it showed that Cre/BW was correlated with diabetes (HR: 0.13; 95% CI: 0.10–0.16, *p* < 0.0001). However, on the right side of the inflection point, their relationship gradually became saturated (HR: 0.62; 95% CI: 0.46–0.82, *p* = 0.0008). Furthermore, the inflection point of Cre/BW was 1.06 (log‐likelihood ratio test *p* < 0.001) in males and 0.87 (log‐likelihood ratio test *p* < 0.001) in females. It also found the effect sizes on both sides of the inflection point are not consistent in different sexes. (Table 4).

**TABLE 4:** The result of the two‐piecewise linear regression model

	Male (HR, 95% CI, *p*)	Female (HR, 95% CI, *p*)	Total (HR, 95% CI, *p*)
Fitting model by standard linear regression	0.26 (0.21, 0.31) <0.0001	0.30 (0.22, 0.41) <0.0001	0.27 (0.23, 0.31) <0.0001
Fitting model by two‐piecewise linear regression			
Inflection point of Cre/BW	1.06	0.87	1.06
≤ Inflection point	0.11 (0.08, 0.16) <0.0001	0.05 (0.02, 0.12) <0.0001	0.13 (0.10, 0.16) <0.0001
> Inflection point	0.55 (0.40, 0.75) 0.0002	0.59 (0.39, 0.89) 0.0126	0.62 (0.46, 0.82) 0.0008
*p* for log likelihood ratio test	<0.001	<0.001	<0.001

*Note*: We adjusted for age, gender, SBP, DBP, FPG, TG, HDL‐C, LDL‐, smoking and drinking status, and family history of diabetes in Total. And adjusted age, SBP, DBP, FPG, TG, HDL‐C, LDL‐C, smoking and drinking status, and family history of diabetes in different gender.

Abbreviations: CI, confidence interval; Cre/BW, creatinine to body weight ratio; DBP, diastolic blood pressure; FPG, fasting plasma glucose; HDL‐C, high‐density lipoprotein cholesterol; HR, hazard ratio; LDL‐C, low‐density lipid cholesterol; Ref, reference; SBP, systolic blood pressure; TG, triglyceride.

### 3.6 Kaplan‐Meier analysis

**FIGURE 2:**
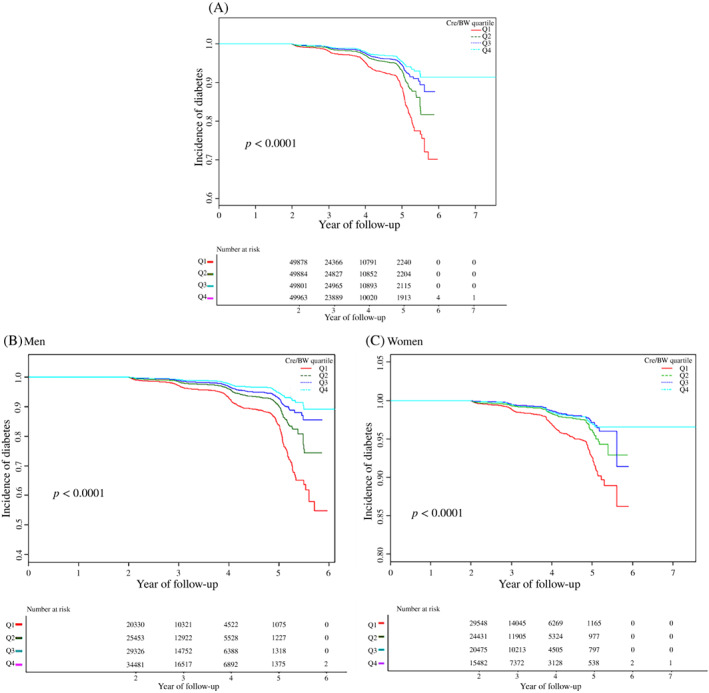
Kaplan–Meier event‐free survival curve. (A) Kaplan–Meier analysis of incidence of diabetes based on Cre/BW quartiles (log‐rank, *p* < 0.0001) in total. (B) Kaplan–Meier analysis of the incidence of diabetes in men. (C) Kaplan–Meier analysis of the incidence of diabetes in women. Abbreviation: Cre/BW, creatinine to body weight ratio

The Kaplan‐Meier curve indicated a significant difference in the risk of diabetes among the quartiles of Cre/BW (log‐rank test, *p* < 0.0001). With the increase of Cre/BW, the accumulated risk of diabetes gradually attenuated, and the relationship between the lowest quartile (Q1) and the risk of diabetes is the strongest. Moreover, it also found statistically significant differences between men and women. (Figure 2).

### 3.7 The results of subgroup analyses

There was a linear correlation between Cre/BW and diabetes in all age subgroups, but the intensity of the relationship was different in different age groups (*p* for trend =0.0004). It suggested that the relationship between Cre/BW and diabetes was affected by age. The results of subgroup and interaction analysis results shown in Table S6.

### 3.8 The predictive value of Cre/BW for diabetes

**FIGURE 3:**
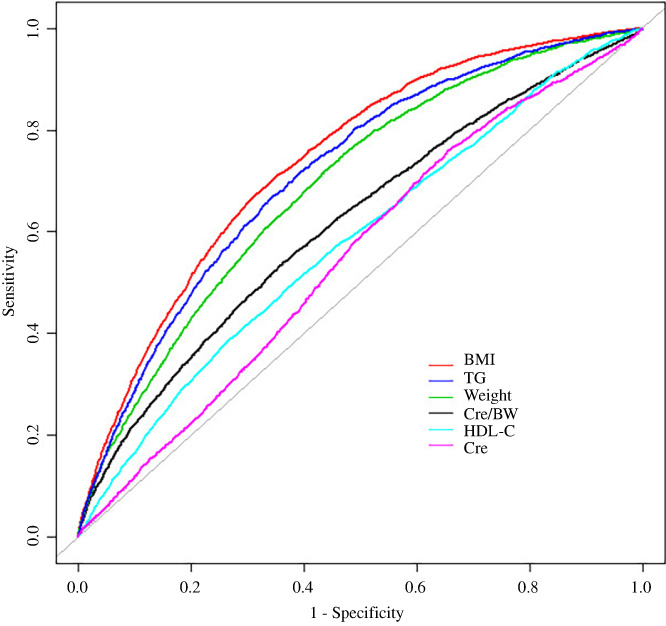
The results of receiver operating characteristics curves. Abbreviations: BMI, body mass index; Cre/BW, creatinine to body weight ratio; HDL‐C, high‐density lipoprotein cholesterol; TG, triglyceride

Figure 3 shows ROC analysis results of Cre/BW, BMI, weight, TG, HDL‐C, and Cre for predicting diabetes. It can be seen that among these markers, Cre/BW has good discrimination ability in predicting the risk of diabetes, with an AUC of 0.6168, whereas BMI seems to be a better predictor of diabetes, with an AUC of 0.7398, sensitivity of 68.20% and specificity of 67.72% (Table 5). Weight and TG also have good predictive performance for DM with AUC of 0.691 and 0.717. In addition, Cre/BW has better predictive performance than Cre (AUC = 0.551).

**TABLE 5:** Effect of Cre/BW, BMI, weight, TG, HDL‐C, and Cre for the risk of diabetes

Variable	AUC (95% CI)	Cutoff point	Specificity	Sensitivity
Cre/BW	0.617 (0.6078–0.626)	1.004	0.637	0.538
BMI	0.740 (0.733–0.747)	24.595	0.677	0.682
Weight	0.691 (0.683–0.699)	65.050	0.555	0.727
TG	0.717 (0.710–0.724)	1.255	0.610	0.715
HDL‐C	0.581 (0.570–0.592)	1.175	0.733	0.387
Cre	0.552 (0.543–0.560)	61.956	0.345	0.759

Abbreviations: AUC, area under the curve; BMI, body mass index; Cre, serum creatinine; Cre/BW, creatinine to body weight ratio; HDL‐C, high‐density lipoprotein cholesterol; TG, triglyceride.

## 4 DISCUSSION

After dealing with the missing value, this study found that there was a negative correlation between Cre/BW and the incidence of diabetes in the Chinese population, and the effect values were different among different age groups. The correlation remained significantly independent of several confounders such as age, sex, SBP, DBP, FPG, TG, HDL‐C, LDL‐C, smoking and drinking consumption, and family history of diabetes. The relationship between them did not change significantly in the original data, suggesting that their relationship is relatively stable. In addition, Cre/BW has a nonlinear relationship with the incidence of diabetes. Although its predictive value was slightly lower than BMI, weight, and TG, Cre/BW had a better ability to predict the risk of developing diabetes (AUC =0.6168) than Cre alone (AUC = 0.551).

Lin and his colleagues have reported the relationship between Cre/BW and NAFLD,^16^ and it was known that NAFLD is connected with obesity and insulin resistance..^30^ To our knowledge, studies investigating the relevance between Cre/BW and diabetes risks are sparse. Recently, a study conducted by Hashimoto et al.^15^ suggested that an independent association of diabetes risks with Cre/BW ratios in the NAGALA Study in Japan. The results of this study are similar to Hashimoto and his colleagues. After handling missing values, with the increase of Cre/BW, the incidence of diabetes decreased (HR: 0.268; 95% CI: 0.229–0.314), and it also makes sense in different genders (HR: 0.255; 95% CI: 0.212–0.307 for men and HR: 0.297; 95% CI: 0.218–0.406 for women). Moreover, a nonlinear relationship was found between Cre/BW and incidence of diabetes (after adjusting for age, gender, SBP, DBP, FPG, TG, LDL‐C, HDL‐C, smoking and drinking consumption, and family history of diabetes) by the cubic spline smoothing technique, and the effect sizes on the left and right sides of the inflection point were inconsistent: left (HR: 0.13; 95% CI: 0.10–0.16, *p* < 0.0001); right (HR: 0.62; 95% CI: 0.46–0.82, *p* = 0.0008). This result suggested there was a saturation effect between Cre/BW and incidence of diabetes.

Under ideal conditions, Cre is considered a good substitute for skeletal muscle.^31^ Abnormal muscle protein metabolism and skeletal muscle atrophy have been reported in T2DM patients.^7^, ^13^ When weight gain is caused by decreased muscle mass and an increase fat mass, especially visceral fat accumulation,^32^ the Cre/BW is a more reliable skeletal muscle mass indicator than Cre or weight alone.^16^, ^33^ The relationship between weight‐adjusted Cre and metabolic parameters is better than height‐adjusted Cre,^34^ and it is closely related to MS^35^ and NAFLD. Therefore, although Cre/BW was not as predictive as BMI, it combined with Cre and body weight takes into account more factors, such as weight gain associated with muscle loss. This is the first time it has been compared with other indicators, the results may help to develop diabetes prediction strategies in the future. The mechanism underlying the association between the reduction of skeletal muscle mass and the occurrence of diabetes has not been cleared, but it may be multiple mechanisms as follow. Skeletal muscle plays a vital role in glucose metabolism. It is one of the main parts of insulin‐mediated glucose uptake, especially postprandial glucose.^36^ Accompanied by decreased skeletal muscle mass, decreased insulin sensitivity, abnormal glucose, and fatty acid metabolism, the maintenance and increase of skeletal muscle mass may ameliorate insulin resistance.^12^, ^37^ The mitochondrial network in muscle cells maintains the movement and metabolic functions of skeletal muscle. The decrease in skeletal muscle volume leads to the decline of mitochondria, which leads to the inability to metabolize fatty acids in skeletal muscle, which may increase insulin resistance by inhibiting insulin signaling, including inhibition of glucose transporter type 4 (GLUT4).^38^, ^39^, ^40^ The tissue‐specific knockout of glucose transporter 4 (GLUT4) in muscle showed severely impaired glucose tolerance and hyperinsulinemia.^41^ Besides, nuclear factor secreted by skeletal muscle has been found to prevent insulin resistance.^9^, ^42^ Therefore, insulin resistance may be a potential mechanism linking muscle mass and diabetes, and weight‐adjusted Cre may be a good representative parameter of diabetes risk.

One of the key strengths of our study was a relatively large sample size and a multicenter study. Furthermore, this study found a nonlinear relationship between Cre/BW and diabetes risk and further explored this. Moreover, in order to maximize statistical power and minimize bias, the study dealt with missing values, and sensitivity analysis was performed to evaluate the possible influence of missing values. At the same time, the target independent variable is taken as a continuous variable and a classified variable, respectively. This method can enhance the robustness of data analysis.

However, several limitations need to be mentioned in the present study. First, because the study population is limited to Chinese participants, this may not be generalized to other populations. Second, because the primary research was not designed to discuss the relationship between Cre/BW and diabetes, there is inevitably a lack of data. However, the study handled the missing data, and the sensitivity analysis indicated that the nonmissing data was consistent with the processed data, and the result was not affected. Third, this study was based on the secondary analysis of published data, and some variables cannot be analyzed in this study, such as exercise, body fat mass, and insulin resistance. Fourth, because there is no oral glucose tolerance test in the study population, this may underestimate the incidence of diabetes. However, oral glucose tolerance testing is impractical in a large sample population.

## 5 CONCLUSION

This study demonstrated that Cre/BW had a negative correlation with the risk of diabetes in Chinese adults, and this relationship was nonlinear. Adults with lower Cre/BW should be alerted to the risk of diabetes and early screening should be carried out for those with risk factors for diabetes.

## DISCLOSURE

The authors declare no conflict of interests.

## Supporting information


**Appendix S1** Supporting Information
