# Supplementary material for: Relationship between creatinine to body weight ratios and diabetes mellitus: A Chinese cohort study
Source: J Diabetes. 2022 Jan 10;14(3):167–78. doi: 10.1111/1753-0407.13248 (PMC9060036; doi:10.1111/1753-0407.13248)
Supplement: Supplementary file 1 — Appendix S1 Supporting Information [file JDB-14-167-s001.docx]

**Table S1** Baseline characteristics of study participants in different gender

| Men | Q1(≤0.96) | Q2(0.96 to ≤1.05) | Q3(1.05 to ≤1.19) | Q4(>1.19) | P-value |
| --- | --- | --- | --- | --- | --- |
| AGE(years) | 41.75 ± 11.69 | 42.56 ± 12.18 | 42.43 ± 12.71 | 42.45 ± 14.10 | <0.001 |
| Height(cm) | 174.33 ± 6.16 | 172.69 ± 6.01 | 171.53 ± 5.96 | 169.74 ± 6.00 | <0.001 |
| Weight(kg) | 82.93 ± 10.47 | 74.92 ± 8.04 | 70.03 ± 7.54 | 63.57 ± 7.54 | <0.001 |
| BMI(kg/m^2^) | 27.28 ± 3.09 | 25.13 ± 2.53 | 23.82 ± 2.49 | 22.08 ± 2.57 | <0.001 |
| SBP(mmHg) | 126.89 ± 15.40 | 123.73 ± 15.14 | 122.00 ± 14.93 | 120.25 ± 15.56 | <0.001 |
| DBP(mmHg) | 79.49 ± 11.09 | 77.45 ± 10.55 | 76.23 ± 10.33 | 74.61 ± 10.13 | <0.001 |
| FPG(mmol/L) | 4.86 ± 0.91 | 4.79 ± 0.89 | 4.72 ± 0.89 | 4.62 ± 0.87 | <0.001 |
| TC(mmol/L) | 5.07 ± 0.65 | 5.00 ± 0.63 | 4.96 ± 0.62 | 4.92 ± 0.61 | <0.001 |
| TG(mmol/L) | 1.96 ± 1.52 | 1.71 ± 1.21 | 1.54 ± 1.08 | 1.31 ± 0.90 | <0.001 |
| HDL-C(mmol/L) | 1.23 ± 0.28 | 1.26 ± 0.27 | 1.29 ± 0.28 | 1.33 ± 0.27 | <0.001 |
| LDL-C(mmol/L) | 2.85 ± 0.69 | 2.82 ± 0.67 | 2.79 ± 0.67 | 2.73 ± 0.66 | <0.001 |
| Creatinine(umol/L) | 69.24 ± 8.25 | 75.66 ± 8.10 | 80.53 ± 8.62 | 88.05 ± 10.35 | <0.001 |
| Smoking status |  |  |  |  | <0.001 |
| Never smoker | 3763 (56.55%) | 5272 (60.90%) | 6427 (64.08%) | 8018 (67.62%) |  |
| Ever smoker | 497 (7.47%) | 582 (6.72%) | 667 (6.65%) | 691 (5.83%) |  |
| Current smoker | 2394 (35.98%) | 2803 (32.38%) | 2935 (29.27%) | 3149 (26.56%) |  |
| Drinking status |  |  |  |  | <0.001 |
| Never drinker | 4807 (72.24%) | 6390 (73.81%) | 7448 (74.26%) | 9209 (77.66%) |  |
| Ever drinker | 1564 (23.50%) | 1936 (22.36%) | 2233 (22.27%) | 2356 (19.87%) |  |
| Current drinker | 283 (4.25%) | 331 (3.82%) | 348 (3.47%) | 293 (2.47%) |  |
| Family history of diabetes |  |  |  |  | <0.001 |
| NO | 19977 (98.26%) | 25074 (98.51%) | 28905 (98.56%) | 34049 (98.75%) |  |
| YES | 353 (1.74%) | 379 (1.49%) | 421 (1.44%) | 432 (1.25%) |  |

| Women | Q1(≤0.91) | Q2(0.91 to ≤1.05) | Q3(1.05 to ≤1.19) | Q4(>1.19) | P-value |
| --- | --- | --- | --- | --- | --- |
| AGE(years) | 42.79 ± 11.67 | 41.66 ± 11.89 | 41.32 ± 12.53 | 41.87 ± 14.06 | <0.001 |
| Height(cm) | 161.29 ± 5.64 | 160.31 ± 5.51 | 159.60 ± 5.47 | 158.33 ± 5.61 | <0.001 |
| Weight(kg) | 62.79 ± 8.28 | 56.27 ± 6.04 | 53.20 ± 5.63 | 49.94 ± 5.49 | <0.001 |
| BMI(kg/m^2^) | 24.16 ± 3.18 | 21.92 ± 2.43 | 20.91 ± 2.27 | 19.94 ± 2.21 | <0.001 |
| SBP(mmHg) | 117.59 ± 16.70 | 113.66 ± 15.79 | 112.31 ± 15.49 | 112.48 ± 16.53 | <0.001 |
| DBP(mmHg) | 73.02 ± 10.71 | 70.82 ± 10.06 | 70.06 ± 9.76 | 69.67 ± 9.90 | <0.001 |
| FPG(mmol/L) | 4.74 ± 0.92 | 4.67 ± 0.90 | 4.66 ± 0.90 | 4.68 ± 0.91 | <0.001 |
| TC(mmol/L) | 4.93 ± 0.59 | 4.83 ± 0.57 | 4.78 ± 0.58 | 4.77 ± 0.58 | <0.001 |
| TG(mmol/L) | 1.21 ± 0.86 | 1.02 ± 0.67 | 0.96 ± 0.63 | 0.93 ± 0.60 | <0.001 |
| HDL-C(mmol/L) | 1.42 ± 0.30 | 1.48 ± 0.30 | 1.51 ± 0.31 | 1.52 ± 0.31 | <0.001 |
| LDL-C(mmol/L) | 2.78 ± 0.70 | 2.73 ± 0.69 | 2.72 ± 0.69 | 2.72 ± 0.68 | <0.001 |
| Creatinine(umol/L) | 51.25 ± 6.30 | 56.62 ± 6.12 | 60.97 ± 6.56 | 68.18 ± 8.45 | <0.001 |
| Smoking status |  |  |  |  | 0.313 |
| Never smoker | 6690 (99.72%) | 5591 (99.79%) | 4515 (99.69%) | 3266 (99.85%) |  |
| Ever smoker | 8 (0.12%) | 8 (0.14%) | 6 (0.13%) | 0 (0.00%) |  |
| Current smoker | 11 (0.16%) | 4 (0.07%) | 8 (0.18%) | 5 (0.15%) |  |
| Drinking status |  |  |  |  | 0.104 |
| Never drinker | 6506 (96.97%) | 5436 (97.02%) | 4417 (97.53%) | 3203 (97.92%) |  |
| Ever drinker | 194 (2.89%) | 158 (2.82%) | 108 (2.38%) | 64 (1.96%) |  |
| Current drinker | 9 (0.13%) | 9 (0.16%) | 4 (0.09%) | 4 (0.12%) |  |
| Family history of diabetes |  |  |  |  | <0.001 |
| NO | 28527 (96.54%) | 23709 (97.04%) | 19937 (97.37%) | 15156 (97.89%) |  |
| YES | 1021 (3.46%) | 722 (2.96%) | 538 (2.63%) | 326 (2.11%) |  |

Values are n(%) or mean±SD

BMI, body mass index; SBP, Systolic blood pressure; DBP, Diastolic blood pressure; FPG, fasting plasma glucose; TG, Triglyceride; HDL-C, high-density lipoprotein cholesterol; TC, Total cholesterol; LDL-C, Low-density lipid cholesterol;Cre, Serum creatinine

**Table S2** The description of missing data.

| Variables | Non-missing | Missing |
| --- | --- | --- |
| Age | 199526 | 0 |
| Gender | 199526 | 0 |
| Height | 199526 | 0 |
| Weight | 199526 | 0 |
| BMI | 199526 | 0 |
| SBP | 199506 | 20 |
| DBP | 199505 | 21 |
| TC | 196429 | 3097 |
| FPG | 199526 | 0 |
| TG | 196424 | 3102 |
| HDL-C | 115217 | 84309 |
| LDL-C | 116327 | 83199 |
| Smoking status | 57310 | 142216 |
| Drinking status | 57310 | 142216 |
| Family history of diabetes | 199526 | 0 |

BMI, body mass index; SBP, Systolic blood pressure; DBP, Diastolic blood pressure; FPG, fasting plasma glucose; TG, Triglyceride; HDL-C, high-density lipoprotein cholesterol; TC, Total cholesterol; LDL-C, Low-density lipid cholesterol

**Table S3** Sensitivity comparative analysis between original and complete data

|  | Original data | complete data | P-value |
| --- | --- | --- | --- |
| N | 199526 | 32495 |  |
| AGE(years) | 42.18 ± 12.65 | 43.14 ± 12.58 | <0.001 |
| GENDER |  |  | <0.001 |
| Male | 109590 (54.93%) | 21093 (64.91%) |  |
| Female | 89936 (45.07%) | 11402 (35.09%) |  |
| Height(cm) | 166.52 ± 8.32 | 167.35 ± 8.23 | <0.001 |
| Weight(kg) | 64.81 ± 12.21 | 66.33 ± 12.12 | <0.001 |
| BMI(kg/m^2^) | 23.26 ± 3.33 | 23.57 ± 3.29 | <0.001 |
| SBP(mmHg) | 119.01 ± 16.37 | 119.79 ± 15.83 | <0.001 |
| DBP(mmHg) | 74.16 ± 10.80 | 74.96 ± 10.50 | <0.001 |
| FPG(mmol/L) | 4.91 ± 0.61 | 4.97 ± 0.62 | <0.001 |
| TC(mmol/L) | 4.71 ± 0.90 | 4.78 ± 0.89 | <0.001 |
| TG(mmol/L) | 1.34 ± 1.04 | 1.45 ± 1.09 | <0.001 |
| HDL-C(mmol/L) | 1.37 ± 0.31 | 1.34 ± 0.31 | <0.001 |
| LDL-C(mmol/L) | 2.77 ± 0.68 | 2.74 ± 0.69 | <0.001 |
| Cre(umol/L) | 69.83 ± 14.98 | 72.05 ± 14.94 | <0.001 |
| Smoking status |  |  | 0.024 |
| Never smoker | 43542 (75.98%) | 24547 (75.54%) |  |
| Ever smoker | 2459 (4.29%) | 1322 (4.07%) |  |
| Current smoker | 11309 (19.73%) | 6626 (20.39%) |  |
| Drinking status |  |  | <0.001 |
| Never drinker | 47416 (82.74%) | 26121 (80.38%) |  |
| Ever drinker | 8613 (15.03%) | 5505 (16.94%) |  |
| Current drinker | 1281 (2.24%) | 869 (2.67%) |  |
| Family history of diabetes |  |  | <0.001 |
| NO | 195334 (97.90%) | 30662 (94.36%) |  |
| YES | 4192 (2.10%) | 1833 (5.64%) |  |

BMI, body mass index; SBP, Systolic blood pressure; DBP, Diastolic blood pressure; FPG, fasting plasma glucose; TG, Triglyceride; HDL-C, high-density lipoprotein cholesterol; TC, Total cholesterol; LDL-C, Low-density lipid cholesterol; Cre, Serum creatinine

**Table S4** Sensitivity comparative analysis between pre-imputation and post-imputation.

|  | original data | imputation 1 | imputation 2 | imputation 3 | imputation 4 | imputation 5 | P-value |
| --- | --- | --- | --- | --- | --- | --- | --- |
| Age | 42.18 ± 12.65 | 42.18 ± 12.65 | 42.18 ± 12.65 | 42.18 ± 12.65 | 42.18 ± 12.65 | 42.18 ± 12.65 | 1.000 |
| SBP | 119.01 ± 16.37 | 119.01 ± 16.37 | 119.01 ± 16.37 | 119.01 ± 16.37 | 119.01 ± 16.37 | 119.01 ± 16.37 | 1.000 |
| DBP | 74.16 ± 10.80 | 74.16 ± 10.80 | 74.16 ± 10.80 | 74.16 ± 10.80 | 74.16 ± 10.80 | 74.16 ± 10.80 | 1.000 |
| FPG | 4.91 ± 0.61 | 4.91 ± 0.61 | 4.91 ± 0.61 | 4.91 ± 0.61 | 4.91 ± 0.61 | 4.91 ± 0.61 | 1.000 |
| TG | 1.34 ± 1.04 | 1.34 ± 1.03 | 1.34 ± 1.03 | 1.34 ± 1.03 | 1.34 ± 1.03 | 1.34 ± 1.03 | 0.701 |
| HDL-C | 1.37 ± 0.31 | 0.80 ± 0.71 | 0.80 ± 0.71 | 0.80 ± 0.71 | 0.80 ± 0.71 | 0.80 ± 0.71 | <0.001 |
| LDL-C | 2.77 ± 0.68 | 1.62 ± 1.46 | 1.62 ± 1.46 | 1.62 ± 1.46 | 1.62 ± 1.46 | 1.62 ± 1.46 | <0.001 |

SBP, Systolic blood pressure; DBP, Diastolic blood pressure; FPG, fasting plasma glucose; TG, Triglyceride; HDL-C, high-density lipoprotein cholesterol; LDL-C, Low-density lipid cholesterol

**Table S5** Collinearity diagnostics steps

|  | Step 1 | Step 2 | Step 3 | Step 4 |
| --- | --- | --- | --- | --- |
| AGE | 1.4 | 1.3 | 1.3 | 1.3 |
| GENDER | 3.5 | 3.4 | 3.4 | 3.4 |
| BMI | 116.6 | 15.9 | 2.2 | 2.2 |
| SBP | 2 | 2 | 2 | 2 |
| DBP | 1.9 | 1.9 | 1.9 | 1.9 |
| FPG | 1.1 | 1.1 | 1.1 | 1.1 |
| TC | 6.9 | 6.9 | 6.9 | NA |
| TG | 1.8 | 1.8 | 1.8 | 1.3 |
| HDL-C | 1.5 | 1.5 | 1.5 | 1.2 |
| LDL-C | 5.6 | 5.6 | 5.6 | 1.1 |
| Cre/BW | 32.1 | 32 | 2 | 2 |
| Smoking status | 1.3 | 1.3 | 1.3 | 1.3 |
| Drinking status | 1.1 | 1.1 | 1.1 | 1.1 |
| Family history of diabetes | 1 | 1 | 1 | 1 |

VIF = 1/(1-R^2^).

Cre/BW, creatinine to body weight ratio; BMI, body mass index; SBP, Systolic blood pressure; DBP, Diastolic blood pressure; FPG, fasting plasma glucose; TG, Triglyceride; HDL-C, high-density lipoprotein cholesterol; TC, Total cholesterol; LDL-C, Low-density lipid cholesterol

**Table S6** Effect size of Cre/BW on diabetes in [different](javascript:;) [age](javascript:;) subgroups

| Characteristic | No of participants | Event(n) | Effect size(HR,95%CI,P) P for interacion |
| --- | --- | --- | --- |
| Age(years)  20 to <30  30 to <40  40 to <50  50 to <60  60 to <70  ≥70 | 26585  77949  42846  28378  16940  6828 | 70  504  748  1168  945  540 | 0.0004  0.001 (0.001, 0.024) <0.0001  0.078 (0.023, 0.262) <0.0001  0.076 (0.028, 0.203) <0.0001  0.294 (0.144, 0.603) 0.0008  0.173 (0.068, 0.437) 0.0002  0.543 (0.225, 1.314) 0.1757 |

The model adjusted for ~~age,~~ gender, SBP, DBP, FPG, TG, HDL-C, LDL-C, smoking and drinking status, family history of diabetes.

SBP, Systolic blood pressure; DBP, Diastolic blood pressure; FPG, fasting plasma glucose; TG, Triglyceride; HDL-C, high-density lipoprotein cholesterol; LDL-C, Low-density lipid cholesterol;
